# Multiomics Topic Modeling for Breast Cancer Classification

**DOI:** 10.3390/cancers14051150

**Published:** 2022-02-23

**Authors:** Filippo Valle, Matteo Osella, Michele Caselle

**Affiliations:** Physics Department, University of Turin and INFN, via P. Giuria 1, 10125 Turin, Italy; mosella@to.infn.it (M.O.); caselle@to.infn.it (M.C.)

**Keywords:** miRNAs, miRNA expression regulation, topic modeling, stochastic block modeling, multiomics, chr14q32

## Abstract

**Simple Summary:**

Topic models are algorithms introduced for discovering hidden topics or latent variables in large, unstructured text corpora. Leveraging on analogies between texts and gene expression profiles, these algorithms can be used to find structures in expression data. This work presents an application of topic modeling techniques for the identification of breast cancer subtypes. In particular, we extended a specific class of topic models to allow a multiomics approach. As an illustrative example, considering both messenger RNA and microRNA expression levels, we were able to clearly distinguish healthy from tumor samples as well as the different breast cancer subtypes. The integration of different layers of information is crucial for the observed classification accuracy. Our approach naturally provides the genes and the microRNAs associated to the specific topics that are used for sample organization. We show that indeed these topics often contain genes involved in breast cancer development and are associated to different survival probabilities.

**Abstract:**

The integration of transcriptional data with other layers of information, such as the post-transcriptional regulation mediated by microRNAs, can be crucial to identify the driver genes and the subtypes of complex and heterogeneous diseases such as cancer. This paper presents an approach based on topic modeling to accomplish this integration task. More specifically, we show how an algorithm based on a hierarchical version of stochastic block modeling can be naturally extended to integrate any combination of ’omics data. We test this approach on breast cancer samples from the TCGA database, integrating data on messenger RNA, microRNAs, and copy number variations. We show that the inclusion of the microRNA layer significantly improves the accuracy of subtype classification. Moreover, some of the hidden structures or “topics” that the algorithm extracts actually correspond to genes and microRNAs involved in breast cancer development and are associated to the survival probability.

## 1. Introduction

A crucial problem in modern computational biology is the integration of different sources of information in the framework of the so-called “precision medicine” [1]. Thanks to the impressive improvement of experimental techniques and the creation of dedicated databases, plenty of different ’omics datasets are available. However, these datasets are difficult to integrate in a coherent picture. They are typically noisy and sparse; they can strongly depend on experimental and processing choices and biases, such as normalization or imputation techniques, and present different constraints—for example, due to (often unknown) specific regulatory interactions. At the same time, only by combining different layers of information can we hope to understand complex pathologies such as cancer and, thus, optimize the therapeutic protocols. In fact, a major goal would be to be able to identify as soon as possible the particular cancer subtype of a given patient, find the corresponding drivers and altered pathways, and thus, possibly, fine-tune the therapy. A fundamental preliminary step is the development of algorithms able to identify and extract the relevant structure and organization of tumor samples using the different available layers of molecular information.

In particular, topic modeling has been recently proposed as a computational technique to identify hidden structures in gene expression data [2,3]. Topic models are a set of algorithms originally developed to extract latent variables from text corpora [4,5,6]. The most popular of these algorithms is the so-called Latent Dirichlet Allocation [5] (LDA), which has been successfully applied not only in texts analysis, but also in other contexts such as bioinformatics [7].

LDA is based on the assumption of a Dirichlet prior for the latent variables. This choice simplifies the statistical inference problem making the algorithm highly efficient. However, many complex systems in which LDA is applied, including expression data, are characterized by the emergence of power-law distributions, which are very far from the Dirichlet assumption [8,9,10,11]. Moreover, the optimal number of topics must be identified by the user in the standard LDA formulation [5].

To overcome these problems, a new class of algorithms based on hierarchical Stochastic Block Modeling (hSBM) was recently proposed [10]. These algorithms are based on the formal equivalence between the topic identification problem and the community detection problem in bipartite networks [12,13,14], where well-developed techniques based on stochastic block modeling [15] can be applied without the need of a Dirichlet prior.

We recently performed a comparative study [3] of different topic modeling algorithms on the task of identifying cancer subtypes from breast and lung cancer gene expression datasets from The Cancer Genome Atlas (TCGA) [16,17]. We found that hSBM typically outperforms other algorithms in the clustering task. Importantly, this algorithm presents the additional advantages of naturally selecting the number of clusters and of providing the genes significantly associated with the latent structure on which the classification is based. We were able to show that the established cancer subtype organization for both breast and lung cancer was well-reconstructed by the latent topic structure inferred by hSBM and that the topic content itself was very informative. In fact, topics associated with specific cancer subtypes were enriched in genes known to play a role in the corresponding disease, and were related to the survival probability of patients.

This paper extends our previous study by integrating in the hSBM framework multiple layers of information. While the integration of additional biological information should generally improve the accuracy of the statistical inference, it is important to stress that this is not always trivially true. Highly noisy or irrelevant data layers could interfere with the task. We will show an empirical example of such a negative interference. Therefore, the addition of new layers should be driven by a clear biological motivation.

We will focus on the illustrative case of breast cancer, which is the most commonly diagnosed cancer type and the leading cause of cancer death in women worldwide [18], with three main goals:

- First, we will show how different layers of biological information can be efficiently integrated in the hSBM framework. We release the python package *nSBM*, inherited from hSBM [10], which is ready to install, easily executable, and can be used to infer the topic structure starting from different layers and types of biological data.
- Second, focusing on breast cancer, we will show that the combination of microRNA and protein-coding expression levels greatly improves the algorithm’s ability to identify cancer subtypes. These findings further confirm the important role previously recognized in several studies that miRNAs play in cancer development [19,20].
- Third, we use the inferred topic structure to select a few genes, miRNAs, and chromosomal duplications that seem to have a prognostic role in breast cancer and, thus, could be introduced as additional signatures of specific breast cancer subtypes. The extension of subtype signatures can help clinicians to fine-tune diagnostic protocols in the framework of a precision medicine approach to cancer [1].

## 2. Results

### 2.1. nSBM: A Multibranch Topic Modeling Algorithm

Many real-word networks are accompanied by annotations or metadata describing different node properties. For example, in social networks, information about age, gender, or ethnicity can be associated to the nodes or the data capacity can be associated to the nodes of the Internet network [21]. In a similar way, different ’omics can provide additional information to biological networks. These metadata can improve the performance of community detection algorithms by providing additional levels of node correlations that are not accessible only using a single data source [22,23,24]. Given the relation between community detection and topic modeling [10], a similar improvement is expected also in the detection of latent variables using topic modeling analysis on multiomics datasets. Our first goal is, thus, to extend the topic modeling approach to multiomics data, and to test its performances in a concrete biological problem.

The extension of a network-based topic modeling algorithm to multipartite networks was recently proposed in the classic context of text analysis by [23], and we apply here a similar approach to biological data. In this case, networks are generic n-partite networks that contain nodes of *n* types: sample nodes (i.e., patients), and (n−1) sets of nodes (e.g., protein-coding mRNA levels, microRNAs) that represent different features associated with the sample nodes.

**Figure 1:**
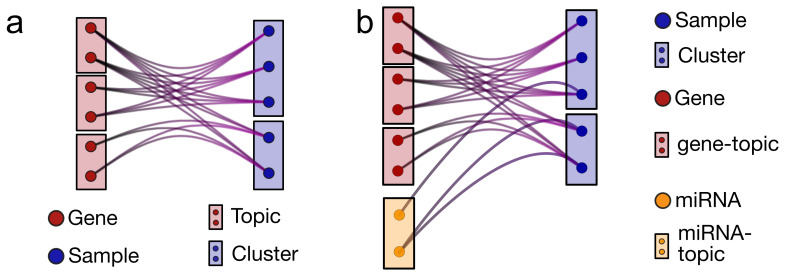
Cartoon of multipartite networks with samples, protein-coding genes, and microRNAs. (**a**) A bipartite network with a layer of protein-coding genes and a layer of samples. A gene is connected to a sample if it is expressed in that sample and the link weight is proportional to the expression level. (**b**) A tripartite network obtained by adding the miRNA expression layer. The topic model algorithm essentially outputs a block or topic structure in each layer.

The topology of the n-partite network is starlike with a center containing the sample nodes and n−1 branches (Figure 1b). Each node in a branch can be connected with all the sample nodes, but no connection exists between nodes within a branch nor between nodes in different branches. This is the natural generalization of the standard bipartite network shown in Figure 1a. In the biological example that will be addressed in the following, only two branches are present: protein-coding genes and microRNAs. However, the presented scheme is general and can be easily extended to several branches at the expense of computational speed. We will discuss the addition of a third sample feature capturing the gene Copy Number Variation (CNV).

We shall denote in the following as “links” the connections between the branch nodes and the sample nodes. Each link is characterized by a weight. The weights can have a different nature depending on the branch. For instance, weights on links connecting the gene branch with the samples encode the expression level (here in FPKM units) and, analogously, the links connecting to miRNAs report the miRNA expression level. When we add a layer with the CNV information, the links are weighted with the number of copies of the gene in the connected sample. The algorithm interprets the weight $w_{ij}$ between node *i* and node *j* as a collection of $w_{ij}$ independent edges. We will use the term “edge” for this elementary unit of link weights.

Once the multipartite network is defined, the statistical inference procedure leading to the topic structure is a straightforward extension of the procedure developed for the hierarchical Stochastic Block Model (hSBM) [10], which we already applied in its bipartite form to expression data [25]. hSBM is a generative model that basically searches the parameters (θ) that maximize the probability that the model describes the data (A)

P(θ|A)∝P(A|θ)P(θ).

The model uses a generative process to build a network given a set of parameters θ. Using a Markov Chain Monte Carlo algorithm, these parameters are optimized in a unsupervised way and the optimization continues until the generated model approximates well the data A. (see [10] and references therein for more details).

The output of the algorithm is a partitioning of nodes or a set of “blocks” of nodes associated to probability distributions. The samples are partitioned into “clusters”, while the blocks of nodes in the branches are essentially the “topics”. Since we are considering several branches, we will have topics of different types, such as gene-topics on the gene expression branch, miRNA-topics on the miRNA branch, CNV-topics on the CNV branch, and so on. We will consider clusters and topics as “hard” blocks (i.e., each sample/gene/miRNA belongs to only one block) and distinct (there are no blocks containing different kind of nodes). However, given its probabilistic nature, the algorithm can be naturally extended to fuzzy clusters.

There are several features that distinguish hSBM, and its nSBM extension introduced here, from other clustering or topic modeling algorithms such as LDA.

- Lack of a parametric prior.
  Thanks to the network-based approach and to the particular way links are used to update the block structure, this class of algorithms does not require a specific parametric assumption for the prior probability distribution of the latent variables. This is a major difference with respect to LDA and makes this class of algorithms particularly suited for biological systems in which long-tail distributions and hierarchical structures are ubiquitous (see the discussion on this point and the comparison with LDA in [3]).
- Probability distributions over latent variables of different types.
  The output of the algorithm is not deterministic but is instead a set of probabilities that associate a sample with latent variables of different types P(gene-topic|sample), P(miRNA-topic|sample) and associate different features to topics, such as P(gene|gene-topic) and P(miRNA|miRNA-topic). P(gene-topic|sample) and P(miRNA-topic|sample) represent the contribution of each miRNA- or gene-topic to each sample. On the other hand, P(gene|gene-topic) and P(miRNA|miRNA-topic) quantify how much each gene or miRNA contributes to a specific topic.
  As we will show in the following, these probability distributions capture relevant properties of the biological system.
- Hierarchical topic structure.
  Blocks and the probability distributions described above are available at different layers of resolution, from few large sets (clusters/gene-topics/miRNA-topics) at low resolution to many small sets at a higher resolution. The specific number of layers and their block composition are found by the algorithm optimization process and are not given as input. Therefore, the datasets can be organized in different ways depending on the resolution of interest. Note that not all possible resolutions are trivially present, as in standard hierarchical clustering.
- Concurrent and separate topic organization of the different network layers.
  Different ’omics have typically different normalization, and the numbers associated to different molecular features have often a very different meaning. A major advantage of nSBM with respect to other algorithms [26,27] is that each layer is independently contributing to the optimization process and a topic organization is given for each layer. Therefore, there is no need to reweight the different layers to balance their contributions since they are kept separate while concurrently contributing to the sample clustering. This makes the model suitable to be applied not only to genomics data, as we will discuss in this paper, but, ideally, to any combination and number of different concurrent ’omics.

### 2.2. Subtype Classification of Breast Cancer Samples

The benchmark task we now focus on to test the performance of nSBM is its ability to cluster breast cancer samples according to their subtype annotation. This is an important task for its clinical relevance, but also because the breast cancer subtype could be dependent on a complex combination of factors, including gene and miRNA expression profiles; thus, the classification could be a good test for nSBM.

Breast cancer is indeed a heterogeneous disease, with wide variations in tumor morphology, molecular characteristics, and clinical response [18,28,29,30]. Notwithstanding this variability, it is one of the few tumors for which there is a widely accepted subtype classification [28,31].

Breast cancer samples are usually divided into five different subtypes: *Luminal A*, *Luminal B*, *Triple-Negative/Basal*, *HER2*, and *Normal-like*. For our tests, we used as a benchmark the TCGABiolinks annotations [32,33], as discussed in the Methods section. These annotations are the result of a rather complex process. On the clinical side, the classification is based on the levels of a few proteins whose presence in the biopsy are usually detected using immunohistochemistry (IHC) assays. In particular, these proteins are two hormone-receptors (estrogen-receptor (ER) and progesterone-receptor (PR)); the Human Epidermal growth factor Receptor 2 (HER2); and Ki-67, which is a nuclear antigen typically expressed by proliferating cells and, thus, is used as an indicator of cancer cell growth. On the gene expression side, the same subtypes can be identified by looking at the expression levels of a set of genes included in the so-called “Prediction Analysis of Microarray (PAM)50” [34]. The agreement between PAM50 results and IHC-based subtyping is, in general, reasonably good but far from being perfect. Indeed, the classification task is made particularly difficult by the heterogeneity of cancer tissues (biopsies may contain relevant portions of healthy tissue) and by the intrinsic variability of gene expression patterns in cancer cell lines.

We recently demonstrated that topic-modeling-based algorithms can achieve satisfactory performances in this classification task by looking at gene expression profiles [3] (and not only of the PAM50 genes), and not relying on the known IHC markers. The advantage of this approach is that it avoids problems and ambiguities in classification due to the stochastic fluctuations of the IHC markers or due to the different inference strategies adopted by PAM50 classifiers (see, for instance, [35] for a recent comparison of the performances of different classifiers in a set of breast cancer classification tasks).

Following this line, one of the goals of our study is to evaluate if the integration of miRNA expression levels (and possibly of other layers of information) can further improve the hSBM results presented in ref. [3].

### 2.3. Integrating microRNA Expression Profiles in a Topic Modeling Analysis

It is, by now, well-established that miRNAs play an important role in several human diseases, particularly in cancer. Accordingly, miRNAs have been proposed as diagnostic biomarkers of human cancers [20,36,37]. This is particularly true for breast cancer, for which several studies have highlighted the prognostic role of miRNAs [38].

Following this line of evidence, we integrated miRNA expression levels with protein-coding mRNA levels using a n=3 version of nSBM (which, in the following, we shall denote as triSBM). In this case, the analysis output, besides the clusters of samples and the topics of genes, will also contain a collection of miRNA-topics.

#### 2.3.1. Including miRNAs in the Topic Modeling Analysis Modifies Both the Sample Clusters and the Gene-Topics

We first tested if the integration of miRNAs has an effect on the partition of samples in clusters and on the topic organization in the gene branch.

**Figure 2:**
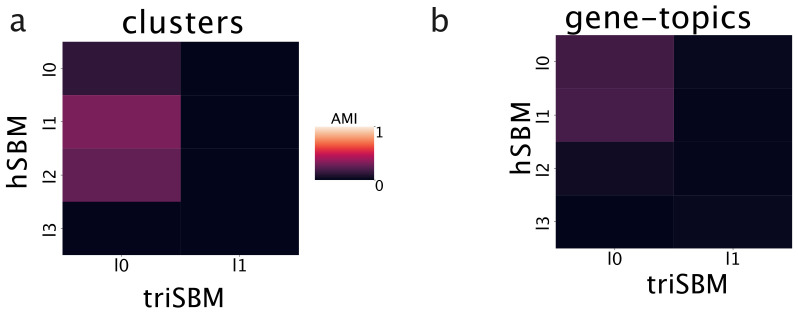
Adding miRNA leads to new topics. The Adjusted Mutual Information between the outputs of triSBM and hSBM (i.e., with and without miRNA). The partitions obtained in output are different for any combination of layers.

Figure 2 reports the Adjusted Mutual Information (AMI) between the partition obtained with a standard hSBM and with triSBM while varying the hierarchy level (l0,l1…), with l0 being the finer layer (the one with smaller sets). We used the AMI to score the overlaps of partitions, since it measures the mutual information between partitions compared with the one obtained by two random partitions. Figure 2a shows that there is a substantial disagreement between the clusters of samples in the two outputs. Similarly, Figure 2b indicates that the same is true for the topics on the protein-coding gene side. The overlap between the partitions obtained by hSBM and triSBM is negligible.

Therefore, the addition of the miRNA branch can radically affect the inferred topic structure and the clustering of samples.

#### 2.3.2. The Inclusion of miRNAs in the Topic Modeling Analysis Leads to a Better Separation of Healthy and Tumor Tissues

**Figure 3:**
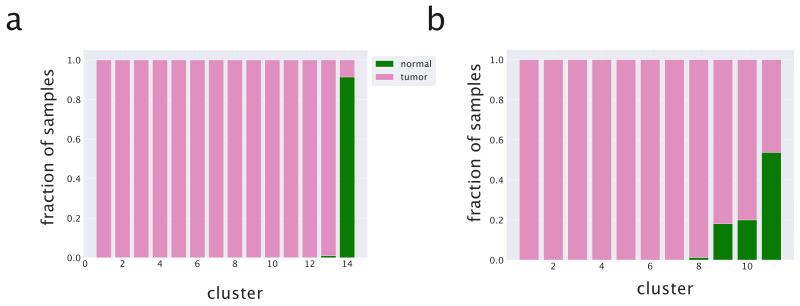
Clustering of breast samples with and without the miRNA branch. We compare normal and solid tumor tissues from TCGA using (**a**) triSBM and (**b**) hSBM at a similar resolution level.

We first tested the ability of the algorithm in recognizing healthy from cancer samples. The hSBM algorithm showed good performances on this task by considering only gene expression data [3], as summarized in Figure 3b. We then tested triSBM, in which gene expression levels were considered jointly with miRNA levels in the same set of TCGA samples. The detailed procedure and the algorithm output at different hierarchical levels are described in the Methods section. We found a significant improvement in the performance of the algorithm. In fact, Figure 3a clearly shows that normal samples are collected in a single cluster by triSBM, while the separation is less neat in the absence of information on miRNA expression (Figure 3b).

**Figure 4:**
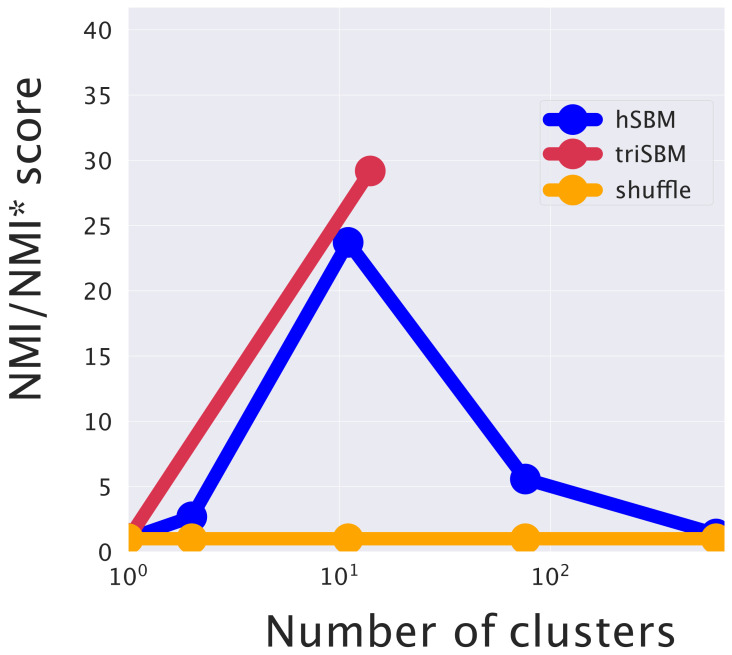
The increase in performance when separating tumor and normal samples by the addition of the miRNA layer. The NMI is evaluated at different resolution levels (numbers of clusters) using (triSBM) or not using (hSBM) the information of miRNA expression. The normal/tumor annotation from TCGA is used as ground truth.

The two model settings (hSBM and triSBM) are compared quantitatively in Figure 4 using Normalized Mutual Information (NMI) as a score [39,40]. The NMI score is explained in detail in the Methods section.

#### 2.3.3. Including miRNAs in the Topic Modeling Analysis Improves the Identification of Cancer Subtypes

**Figure 5:**
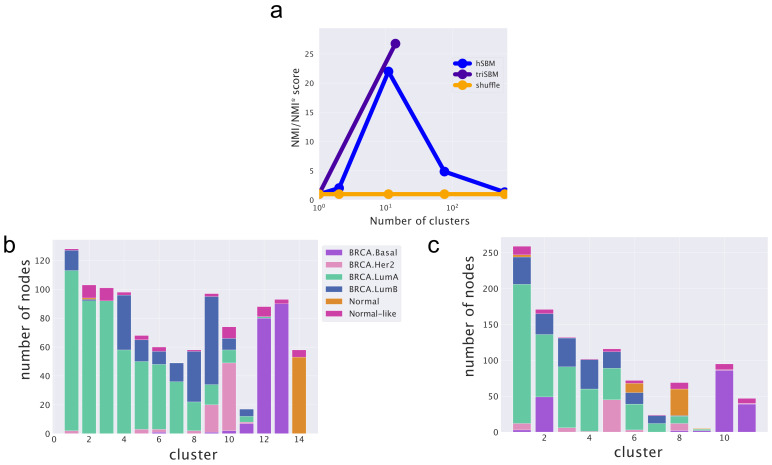
Scores and partitions based on *Subtype_Selected* annotations from [32,33]. (**a**) Scores for both (triSBM and hSBM) setting for the subtype classification problem. (**b**) The miRNA are introduced. We compared the two settings choosing the layers with a compatible number of clusters. (**c**) The clusters from a simple bipartite setting. They are almost similar; in (**c**), Luminal B is identified better. We define Normal as the *Solid Tissue Normal* from TCGA, whilst Normal-Like are the *Primary Tumors* annotated BRCA.Normal from [32].

As a second benchmark, we tested the ability of triSBM to identify breast cancer subtypes. Again, triSBM and hSBM are compared and the results are reported in Figure 5. Further, in this case, the inclusion of miRNA levels improves the algorithm ability to group samples belonging to the same cancer subtype. The improvement is quantified by the NMI scores reported in Figure 5a–c, which show that the improvement is mainly due to the better performance of triSBM in distinguishing LuminalA from LuminalB samples. This was indeed the critical obstacle limiting the performances of hSBM in our previous study [3], suggesting that the distinction of these subtypes crucially depends on miRNA expression levels.

We used the *Subtype Selected* labels provided by TCGABiolinks [32,33] as the ground-truth annotation of subtypes. However, note that this labeling has a less-solid basis with respect to the clear healthy/cancer distinction since the subtypes may not be so clearly defined and can be easily misclassified because of the high tumor heterogeneity.

Note that the standard characterization of breast cancer subtypes relies on the expression level of only few markers. We did not explicitly select these markers in our gene selection process; thus, as previously discussed [3], the emergent sample organization is the result of the global pattern of gene and miRNA expression levels. Therefore, the significant overlap with the standard subtype annotation is highly nontrivial, and the discrepancy does not have to be automatically interpreted as a failure since the standard annotation could be limited.

Given these positive results, we will explore in the following sections the biological information contained in the latent variables inferred by the algorithm and test their possible prognostic role.

#### 2.3.4. Check the Robustness of the Model with an Independent Labeling

We compared the blocks we obtained in output with the annotations of TCGA sample in [41]. First of all, we measured the Adjusted Mutual Information (AMI) between these labels and the Subtype_Selected ones discussed above (AMI is a score between 0 and 1, which measures the mutual information between two annotations compared with the one obtained by comparing two random annotations). We found a value of ∼0.37, which shows that the two labels are not trivially the same and, thus, represent a reliable test of our clusters.

We measured the Normalized Mutual Information score of both the bipartite (hSBM) model and the model that integrates miRNA (triSBM). Results are reported in Supplementary Figure S1. Looking at the figure, we see that our clusters also show a significant agreement (high values of NMI/NMI∗) with this independent classification and, above all, that the agreement improves when including miRNAs.

The overlap between our cluster partition and two independent nonoverlapping labels can be explained by the fact that our partition groups samples at the intersection between the two labeling systems.

#### 2.3.5. Validation on an Independent Source of Data: METABRIC

We applied the same pipeline applied on TCGA to the METABRIC [42] dataset and measured the agreement between our partition on this data and the labels provided by [41]. We confirmed the results obtained on TCGA: the triSBM model has a better agreement (NMI score is reported in Supplementary Figure S2) with the labels assumed as ground truth with respect to the model without miRNA (hSBM).

### 2.4. triSBM Topics Can Be Used to Obtain Subtype-Specific Information

A major advantage of a topic modeling approach to multiomics data is that we can use the information stored in the probability distributions P(topic|sample) to obtain subtype-specific signatures. Following the analysis of [3], we constructed from these probabilities a set of “centered” distributions $\bar{P}\left(\mathrm{topic}|\mathrm{subtype}\right)$ (see the definition in the Equation (5) of the Methods section), which allow us to identify subtype-specific topics (i.e., topics that are particularly enriched in the samples belonging to a particular subtype) that are candidates to play a role in driving the specific features of that subtype.

These topics are nothing but lists of genes and can be investigated using a standard enrichment analysis. The results shown in this paper were computed using the Gene Set Enrichment Analysis GSEA [43] tool. In particular, we concentrated on the keywords extracted from [44,45,46].

We discuss the results of this analysis in the following two subsections.

#### 2.4.1. Analysis of Subtype-Specific Topics of Genes

**Figure 6:**
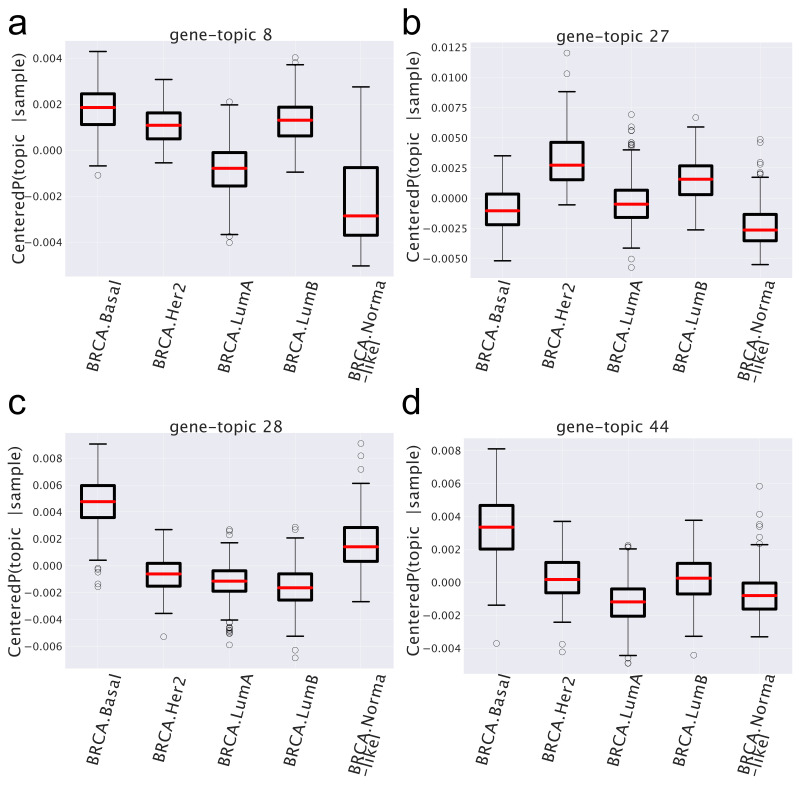
Box plots of the centered P(gene-topic|sample) for different gene-topics. Samples belonging to each subtype may be over- or under-expressed in different topics.

We report in Figure 6 a few examples of $\bar{P}\left(\mathrm{gene}\text{-}\mathrm{topic}|\mathrm{subtype}\right)$ distributions for a few selected topics and in Table 1 the results of the corresponding enrichment analysis.

Looking at the figures and at the table, we see a few interesting patterns.

- There are topics, such as, for instance, topic 8 in Figure 6, which shows a similar behavior in all cancer subtypes and a different one (in the case of topic 8, it is depleted) in the normal tissues. These are the topics that allowed the algorithm to distinguish so accurately normal from cancer samples. In the case at hand, the functional analysis allows to easily understand the reason of this different behavior: the genes contained in topic 8 are strongly enriched in cell cycle keywords, which are likely to be associated to the proliferating nature of tumor tissues.
- Another interesting pattern is well-exemplified by topics 27, 28, and 44 in Figure 6. These are topics that are over-represented only in one particular subtype (in the example, topics 28 and 44 in the Basal subtype and topic 27 in the HER2 one) and can thus be used as signatures of these subtypes. This is in nice agreement with the finding of the gene enrichment analysis, which, for topics 28 and 44, provides a strong enrichment for the keyword SMID_BREAST_CANCER_BASAL_UP, which is known to be associated with the Basal subtype [44], while topic 27 is enriched in the keyword SMID_BREAST_CANCER_ERBB2_UP, which is in fact associated with the HER2 subtype [44]. These topics are the latent variables that allow the algorithm to distinguish among different subtypes.

#### 2.4.2. Analysis of Subtype-Specific Topics of miRNAs

While the above results were similar to the ones already discussed in [3], the novelty of the present analysis is that we can perform a similar study also on the miRNA side. As we will see, this allows for a new independent insight on the problem.

**Figure 7:**
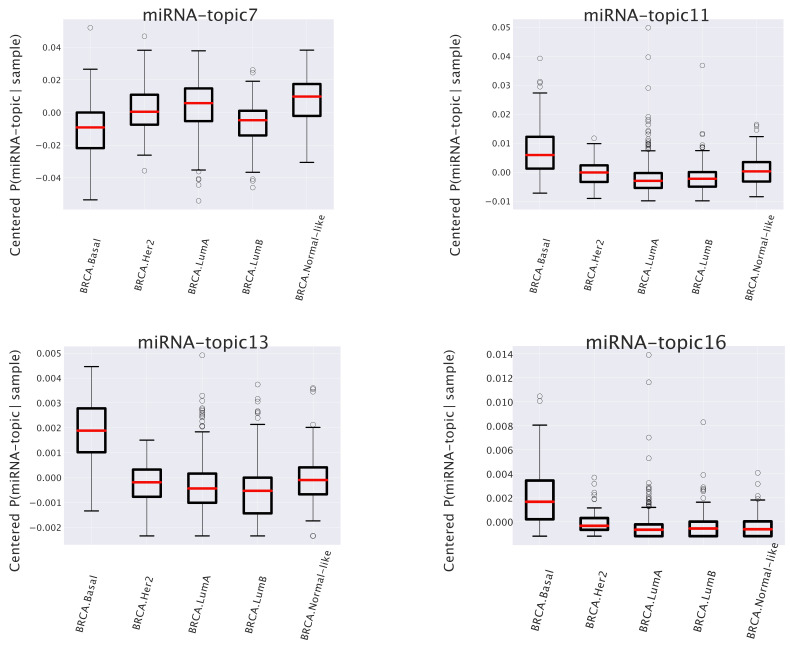
Box plots of the centered P(miRNA-topic|sample). This plot shows that the differences of topic expression in each subtype may be different. Some miRNA-topics are more abundant in samples known to be Basal Subtype.

We report four instances of the $\bar{P}\left(\mathrm{miRNA}\text{-}\mathrm{topic}|\mathrm{subtype}\right)$ probability distributions in Figure 7 and the corresponding enrichment analysis in Table 2. They are, somehow, paradigmatic examples of the type of information that one can obtain from this type of analysis.

**Figure 8:**
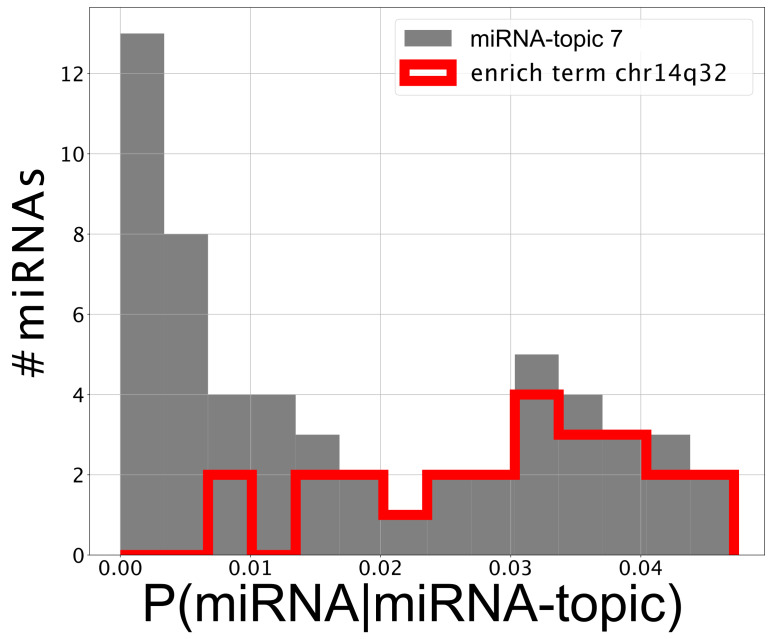
Genes that are annotated in the Gene Set Enrichment Analysis terms contribute more than average to the topic. Contribution of miRNAs to miRNA-topic 7. miRNAs that belong to the ontology specific of locus *c14q32* are highlighted and have high P(miRNA|miRNAs’topic).

- The first one (named miRNA-topic 7 in our output, see https://github.com/BioPhys-Turin/keywordTCGA/blob/main/brca/trisbm/trisbm_level_0_topics.csv, accessed on 10 February 2022) is the typical example of a topic that shows no particular preference for a cancer subtype (see Figure 6) but shows a strong enrichment for a particular chromosomal locus: chr14q32 (see Table 2). This enrichment is due to the fact that most of the miRNAs of the topic are indeed contained in this locus. Moreover, looking at Figure 8, we see that these miRNAs are exactly those with the highest probability to belong to the topic. This strongly suggests that a somatic alteration (duplication or deletion) at this locus could be associated to the onset of cancer and could thus be used as a marker. Indeed, this locus is known to be associated with breast cancer [47]. Accordingly, if we perform a survival analysis between patients with this topic upregulated and patients with the topic downregulated (see next subsection), we find a remarkable increase in the survival probability of patients with the topic downregulated.
  However, this is not the end of the story. Looking at Table 2, we see that the same topic is also enriched in keywords associated to Alzheimer disease. Indeed, it is known that there is a sort of inverse comorbidity [48] between a few types of cancer (in particular, lung [49] and breast [50]) and Alzheimer’s disease. This association is confirmed and supported by our analysis, which also suggests that it could be mediated exactly by the microRNAs contained in miRNA-topic 7. Indeed, some of the miRNAs contained in the topic, such as mir-34c, are known oncosuppressors of breast cancer [51,52] and, at the same time, are recognized markers of Alzheimer’s disease [53,54]. The most important of these is the abovementioned mir-34c, which is in fact, strongly associated with miRNA-topic 7, being the only miRNA in the topic with P(miRNA|miRNA-topic)>0.04 not belonging to the locus chr14q32 (see Figure 8).
- A second class of topics is represented by the other three entries of Figure 7 (miRNA-topics 11, 13, and 16 in our output), which show a different behavior in one of the subtypes with respect to the others (in the present case, these topics are upregulated in samples belonging to the basal subtype). Out of these, only miRNA-topic 11 shows a significant entry in the table of enriched keywords: Table 2. The enrichment is for another chromosomal locus: chr19q13. What is interesting is that this locus has been associated in the past to other types of cancer [55]. Our analysis suggests that it could also play a role in breast cancer and, in particular, in the Basal subtype.

Moreover, we found a nontrivial overlap between genes in these miRNA-topics and the miRNA clusters proposed by [56]. In particular, there were 12 miRNAs in miRNA-topic 7 from cluster cl349_chr14 (estimating the probability of this happening by chance using a hypergeometric test, we obtained a $P\text{-}value≃10^{-5.8}$), and 8 miRNAs in miRNA-topic 11 were assigned with label cl590_chr19 ($P\text{-}value≃10^{-7.4}$).

In the next subsection, we shall study in detail—as an example of the type of analyses that we can perform using the probability distributions obtained from triSBM—the first of these topics.

### 2.5. miRNAs Contained in miRNA-Topic 7 Are Strongly Associated with Breast Cancer and May Affect the Survival of Patients

We can use the information contained in the probability distribution P(miRNA|miRNA-topic) to perform a more refined analysis of the miRNAs contained in the topic. First, we see that 75% of the miRNAs in the topic are annotated with the chr14q32 locus and that they are exactly those with the highest values of P(miRNA|miRNA-topic). This can be visualized in Figure 8, where we highlighted in red the miRNAs annotated to the chr14q32 keyword from GSEA [43].

Then, we sorted the miRNAs on the basis of their value of P(miRNA|miRNAs’topic) and investigated the first ones (see those with P(miRNA|miRNAs’topic)>0.030 in Table 3); it turns out, using the DISEASES tool [57], that most of them are in some way associated with breast cancer. Let us highlight that mir-511, mir-31, and mir-34c are highly important in this miRNA-topic; nevertheless, they do not belong to the c14q32 locus gene set. What is interesting in our analysis is it suggests that these miRNAs, which were studied in the past as separated entities, are most-probably working together. A better understanding of this cooperative behavior could be of great importance to fine-tune future therapeutic protocols. As a first step in this direction, we took advantage of the probabilistic nature of topic modeling to investigate the survival probability of patients.

In particular, since a P(topic|sample) can be assigned to each patient (sample), it is possible to create cohorts of patients based on the importance of a given topic in their transcriptome.

We ran a Cox [58] model to verify which is the contribution of our topic to the survival probability of patients.

**Figure 9:**
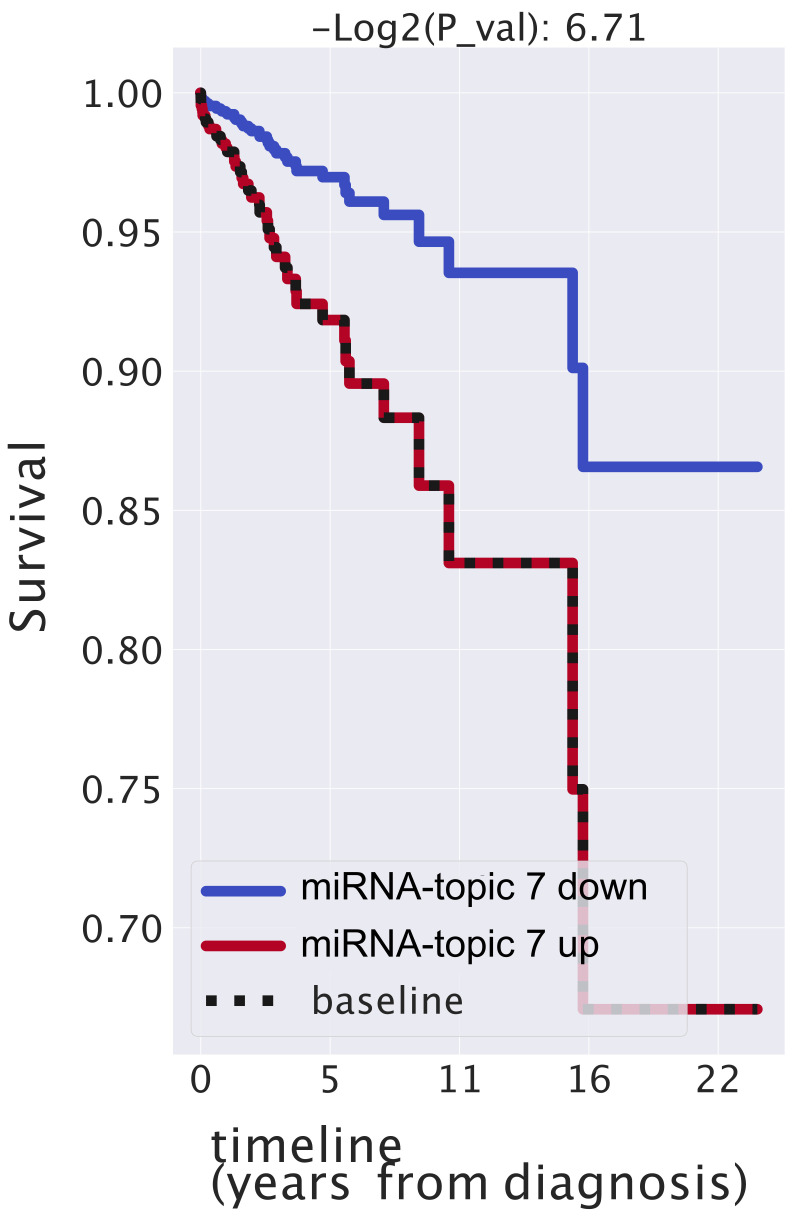
Kaplan–Meier analysis of miRNA-topic 7. We divided patient (samples) into two cohorts using the information regarding the importance of this miRNA-topic in each sample. Patients with a great presence of these topics have smaller values of survival.

We report in Figure 9 the Kaplan–Meyer curves that we obtained.

The contribution of the topic to the survival probability turns out to be very significant: a positive regulation corresponds to higher hazard ratios, meaning that if miRNA inside our topics are expressed higher than normal, the survival probability of patients decreases. While these results should be taken with some caution due to the several sources of bias that may be present in the TCGA population that we tested, it is nevertheless interesting to notice that the presence or absence of this topic has an impact on the survival probability larger than the tumor stage, which is, obviously, strongly correlated with the patient’s prognosis (see Supplementary Figure S3). As a comparison, we also report in Figure S3 variables such as gender (this is not very balanced, as samples are almost all females) or age, which, as expected, do not have significant effects on the survival probability of patients.

Going further in the investigation of the survival probability of the patient, one can wonder if patients in a cluster share a similar prognosis.

If one measures the fraction of patients still alive 3 years after the diagnosis, it is possible to give a prognosis indication of patients in a given cluster. In Figure S7, we reported two clusters in which the prognosis of the patient is significant. In cluster 6, for instance, only 18% of the patients survived more than 3 years. This corresponds to a cluster with a bad prognosis. On the opposite side, more than 60% of patients grouped in cluster 14 survived: we can assert that patients in this set have a favorable prognosis. We measured the significance of these results by comparing the aforementioned percentages to the ones obtained by creating clusters at random (picking up patients from the whole dataset at random 100 times) and obtained significant Z∼3 scores (reported in Figure S7).

## 3. Discussion

There are two main directions in which the analysis discussed in the previous section could be improved. First, one could include in the investigation the regulatory interactions among miRNAs and target genes. Second, one would like to extend the integration to other information layers. We shall discuss in this section a few preliminary attempts in these directions.

### 3.1. Including Regulatory Interactions in the TriSBM Framework

MiRNAs exert their biological function by regulating target genes at the post-transcriptional level. It is thus of great importance to be able to include this information in the topic modeling analysis. This is not an easy task, since miRNAs act in a combinatorial way: typically, several miRNAs cooperate to regulate a single target gene; at the same time, a single miRNA can regulate hundreds of targets. Moreover, while the standard miRNA–target regulatory interaction is of inhibitory type, it sometimes happens that a miRNA can have a widespread (indirect) activatory role by interfering with a repressed epigenetic pathway. These are the so-called “epi-miRNAs” [59,60] that have been recently shown to play an important role in cancer development [60]. Keeping track of these interactions can be of crucial importance to correctly decode the information contained in the miRNA expression data. To this end, one can make use of a few specialized databases of miRNA–target interactions. In particular, in the following, we shall use MirDip [61] and TarBase [62], which are among the most popular ones and are somehow complementary in their target selection choices.

To integrate the regulatory information, we made use of the analogy of this problem with inclusion of the citation information among documents in standard topic modeling applications to texts [23]. In our case, the additional links are not between samples (as it would be a citation link or a hyperlink); therefore, for links between branches in particular, we added gene–miRNA links.

**Figure 10:**
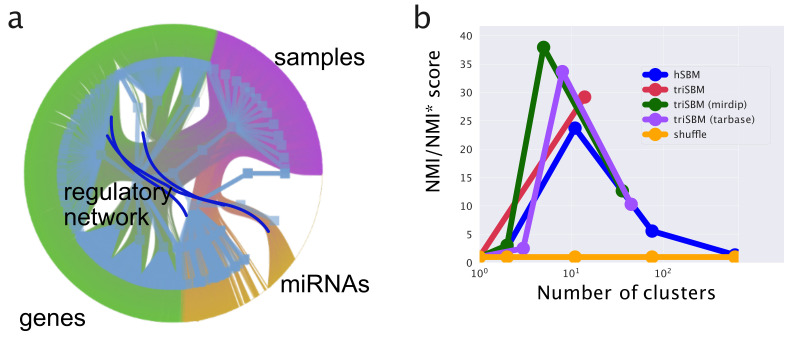
Configuration and scores when adding gene–miRNA links. (**a**) A graphic of a tripartite network with links gene–miRNA. (**b**) The scores of this new setting using two different (mirDIP [61] and TarBase [62]) regulatory networks separately.

We ran the tripartite model as described before; then, in a second moment, we added links gene–miRNA from regulatory network (we tested separately MirDip [61] and TarBase [62]), as shown in Figure 10a. On the fitted triSBM model, we ran steps of the fast merge-split implementation of SBM [63] to improve the description length (see Methods for a precise definition) of the data made by the model, taking advantage of the gene-regulation information in a way similar to the citation between documents when they are used to improve the classification ability of hSBM in that context.

We report in Figure 10b the Normalized Mutual Information, measuring the ability of the full process (fit triSBM, add links, run merge-split) in identifying the breast subtypes. Remarkably enough, we see that by including the information on miRNA–genes interactions, we reach a higher NMI, i.e., a better agreement of our clusters with the subtype organization. This does not happen when simply running merge-split after triSBM is run.

This shows that it is possible to integrate not only multiple layers of sample-related information, but also knowledge about correlations between different kinds of features. Our results represent a first proof of concept in this direction, and we plan to further pursue this type of analysis in future.

### 3.2. Adding Further Layers of Information: The Case of Copy Number Variation

As we discussed in the introduction, the nSBM algorithm can be extended in principle to any other layer of information on the samples. A natural candidate is Copy Number Variation (CNV). It is well-known that chromosomal aberrations are a hallmark of cancer and that several types of cancer are characterized by a well-defined set of chromosomal loci whose deletion or duplication can drive the onset of that particular type of cancer. We already noticed that, using the information contained in the miRNA branch, we could identify two loci whose alteration were known to be associated with the onset of breast cancer. In TCGA database, we also have the information on the CNV values for all samples. We included this information by adding a fourth branch to our algorithm (accordingly, we shall call it in the following, “tetraSBM”). As a preliminary test, we selected only genes with positive CNV (i.e., genes contained in duplicated loci) and that were neglected for the moment deletions.

We performed a gene selection also in this new branch. Highly Copied Genes were selected, keeping the ones with an average (over samples) CNV greater than 3.5. A total 1353 genes passed our selection. This selection would select genes with at least 2 duplications (CNV=4) on average.

It is important to stress that, at this stage nodes, which corresponds with the same gene in the gene expression branch and in the CNV branch, are completely uncorrelated and are seen by the algorithm as independent nodes. We shall discuss below how to address this issue.

In our setting, we have 3000 protein-coding genes in the gene expression branch, 1353 genes in the CNV branch, and 417 of them are represented by nodes in both branches.

We ran the tetraSBM model on this network with samples, protein-coding genes, miRNAs, and CNV genes and obtained two hierarchical levels. In the first one, the four branches were partitioned into 13 clusters, 7 gene-topics, 5 miRNA-topics, and 5 CNV-topics. In the second one, we found 397 clusters, 49 gene-topics, 14 miRNA-topics, and 31 CNV-topics.

Looking at the CNV-topics, we found a very interesting result (see Table 4). Performing the usual Gene Set Enrichment Analysis we found, with very low values of False Discovery Rate (FDR), a few chromosomal loci that we think represent the complete collection of chromosomal aberration associated with breast cancer and could be used as a robust signature of this type of tumor. The relevance of this result is supported by the other set of enriched keywords (taken from [64]), which are reported in Table 4 and show that for some of these loci, the association with breast cancer is already known and is indeed very strong.

On the other side, if we test the performance of tetraSBM to identify the samples subtype, we see that, including the information on CNV, we have a *decrease* in the NMI value (see Supplementary Figure S4). This is not surprising because within the duplicated (or deleted) loci, besides the few drivers of the cancer, there are hundreds of “hitchhikers” genes that simply add noise to the process of subtype classification performed by the other two layers (genes and miRNAs). The variability of the gene expression values that are associated to the different cancer subtypes (and in fact, are allowed to classify the subtypes in the hSBM and triSBM versions of the algorithm) were completely shadowed by the noise induced by the CNV branch. In the Supplementary Figure S5, we reported a bipartite analysis on subtypes with a bipartite network using only the CNV data. This analysis confirms that the CNV layer is less-informative than the layer with only protein-coding genes.

This tells us that adding further layers of information does not automatically improve the quality of clustering. It is always important to perform a careful analysis of the biological information contained in the data and of its possible interference with the other layers. In this particular example, we learned that miRNAs cooperate together to assign coregulated genes to the same gene-topic and samples of the same subtype in the same clusters. This fact becomes particularly clear looking at the probability (see Equation (2) in the Methods section and [65] for further details) of moving nodes between groups: when moving a gene between gene-topics, it is more probable to move in a topic where there are genes with many connections to the miRNAs connected to the gene itself. This is confirmed by the fact that, as we discussed in the previous sections, there are miRNA-topics that overlap with clusters of miRNA [56] known to coexpress in breast cancer. On the other hand, the CNV features force samples with the same duplicated loci to be together and this seems not to be correlated with the cancer subtype, at least in TCGA-BRCA data.

This does not mean that the addition of CNV data is useless. It is only by including CNV that we may have, as we have seen, precise information on the chromosomal aberrations involved in breast cancer. It is also interesting to notice that this information is somehow complementary to the one we obtained in the previous section looking at the miRNA clusters. The chromosomal loci that we detected there are not present in this CNV analysis because their CNV value is below the threshold we fixed to include CNVs in the tetraSBM.

## 4. Materials and Methods

### 4.1. The Cancer Genome Atlas Data

The results published here are, in part, based upon data generated by The Cancer Genome Atlas (TCGA) managed by the NCI and NHGRI. Information about TCGA can be found at https://cancergenome.nih.gov, accessed on 10 February 2022. TCGA data of breast cancer samples were downloaded through portal.gdc.cancer.gov, accessed on 10 February 2022. We selected *TCGA* program, *TCGA-BRCA* Project Id, *transcriptome profiling* as Data Category. We chose *Gene Expression Quantification* and *RNA-Seq* as the Data Type and Experimental Strategy to download gene expression data in HTSeq-FPKM. Moreover, we downloaded the number of reads per million of miRNA mapped from the *miRNA Expression Quantification* Data Type generated with the *miRNA-Seq Experimental Strategy*.

### 4.2. Metadata and Cancer Subtypes

In order to benchmark our results, we compared in Section 2 the clusters of samples obtained by our algorithm with TCGA annotation, which we considered as our “ground truth”. We choose the annotations available through TCGABiolinks [32,33] and, in particular, the one defined as *Subtype_Selected*. Those subtype annotations are provided by [66] and are based on previously published studies [17,67] about breast cancer based on TCGA.

In other analyses, we needed to know if a sample was a primary tumor or derived from normal tissues. Solid Normal Tissues samples are the ones with *sample type* 10 or 11 in TCGA barcode (10 to 19 are normal types) (https://docs.gdc.cancer.gov/Encyclopedia/pages/TCGA_Barcode/, accessed on 10 February 2022).

We downloaded the independent Breast Cancer Consensus Subtypes (BCCS) related to the TCGA files provided by the Supplementary files of [41].

### 4.3. METABRIC miRNA Landscape Data

We downloaded METABRIC data from the European Genome-Phenome archive.

We downloaded METABRIC miRNA landscape study (EGAS00000000122), in particular, Normalized miRNA expression data (EGAD00010000438) and Normalized mRNA expression (EGAD00010000434).

### 4.4. nSBM: A Multibranch Stochastic Block Modeling Algorithm

We collect here some further information on the nSBM algorithm.

- The search for optimal allocation of the latent variables is performed by inheriting and expanding [25] hierarchical Stochastic Block Modeling (hSBM) introduced in [10]. Note that the training process is performed simultaneously in all branches of the network: this means that all the types of data contribute to the learning process at the same time, without, in principle, any preference at the beginning.
- As mentioned in the main text, nSBM attempts to maximize the posterior probability P(θ|A) that the model describes the data
  (1) P(θ|A)∝P(A|θ)P(θ)
  in a completely nonparametric [68] way. Instead of maximizing the probability of the model, as usual, it minimizes the Description Length Σ=−logP(A|θ)−logP(θ). We used the minimise_nested_blockmodel_dl function from graph-tool [69]. In our setting, A is a block matrix in which each block is a “Bag of Features” (i.e., genes, miRNAs, …). It can be seen as a two-dimensional matrix whose entries $w_{ij}$ are the weights mentioned above. The probability of accepting the move of a node with a neighbor *t* from group *r* to group *s* is [65]
  (2) $$P\left(r\to s|t\right)=\frac{e_{ts}+\epsilon }{e_{t}+\epsilon B},$$
  where $e_{ts}$ is the number of edges between groups *t* and *s*; $e_{t}$ is the total number of edges connected to group *t*. From this, another advantage of a multibranch approach should be clear: different ’omics may have their own normalization. In fact, when moving a sample from *r* to *s*, the probability is estimated considering only the branch to which *t* belongs. If the node *t* is a gene, $e_{ts}/e_{t}$ is normalized, taking only into account the mRNA expression values.
- We set the algorithm so as to do a sort of model selection minimizing the Description Length Σ10 times and then choosing the model with the shortest Description Length.
- We used the nested, degree-corrected [68] version of the model [70] so as to obtain in output a hierarchy of results.
- The intrinsic complexity of typical Stochastic Block Modeling algorithms is $O\left(\left(\left(n_{m}+\tau \right)E+V\ln\left(V\right)\right)*\frac{\ln(V)}{\ln(\sigma )}\right)$ (τ, $n_{m}$, and σ are hyperparameters of the model), which equals $O(V\log^{2}V)$ if the graph is sparse (E∼O(V)) [71], where *V* is the number of vertices (samples, genes, and microRNAs) and *E* is the number of edges. If E>>V, the complexity is not logarithmic and the CPU time needed to minimize the description length increases as well. In this case, to reduce the CPU bottleneck, one can apply a log-transformation to the data, which strongly reduces the number of edges *E*. We ran the model on a 48-core machine with 768 GB of memory [72].

In our setting, we have V∼O(1000) vertices, E∼O(1000000) edges, and the network is indeed very dense. In order to reduce the number of nodes and edges, a preprocessing step is needed. We shall discuss this issue in the next subsection.

We considered 1222 samples from TCGA-BRCA project and selected the 1200 with a valid annotation from [32,33]; then, we ran the model on a tripartite network built with normal and tumor samples from TCGA on one branch, 3000 FPKM normalized gene expression data on a second branch and 1300 miRNA-Sequencing data on the third branch. Note that we did not explicitly selected the known breast Cancer markers, our approach to topic model, as already discussed in [3], took into account the whole expression pattern and did not relay only on few specific markers.The output of the tripartite model consisted of two hierarchical levels with 1 and 14 clusters; 11 and 331 topics; and 33 and 47 miRNA-topics on the three branches, respectively. We ran also, as a comparison without miRNAs, hSBM on a bipartite network and obtained levels with 2, 11, 76, 608 clusters and 5, 17, 62, 390 topics across the hierarchy.

As the output of the model, we find the probability distributions P(topic|sample) and P(gene|topic). These probabilities are defined, in terms of entries of the program, as follows:

(3) $$P\left(\mathrm{topic}|\mathrm{sample}\right)=\frac{\mathrm{number}\;\mathrm{of}\;\mathrm{half}\text{-}\mathrm{edges}\;\mathrm{on}\;\mathrm{sample}\;\mathrm{coming}\;\mathrm{from}\;\mathrm{topic}}{\mathrm{number}\;\mathrm{of}\;\mathrm{half}\text{-}\mathrm{edges}\;\mathrm{on}\;\mathrm{sample}}$$

and

(4) $$P\left(\mathrm{gene}|\mathrm{gene}\text{-}\mathrm{topic}\right)=\frac{\mathrm{number}\;\mathrm{of}\;\mathrm{half}\text{-}\mathrm{edges}\;\mathrm{to}\;\mathrm{gene}\text{-}\mathrm{topic}\;\mathrm{going}\;\mathrm{to}\;\mathrm{gene}}{\mathrm{number}\;\mathrm{of}\;\mathrm{half}\text{-}\mathrm{edges}\;\mathrm{to}\;\mathrm{gene}\text{-}\mathrm{topic}}.$$

The same is true for miRNA-topics and for each and every eventual additional layer of features.

### 4.5. Gene and miRNA Selection

The data provided in the atlas consisted of 1222 (∼1100 have both mRNA and miRNA transcript profiles data) samples associated with almost 20,000 genes and 2000 miRNA entries. Without preprocessing, this would have led to an adjacency matrix too big to be handled efficiently by the algorithms.

We performed two kinds of preprocessing to reduce the number of nodes and the number of edges.

In order to reduce the number of nodes, we filtered genes and miRNAs selecting only the highly variable ones. The highly variable are the ones with the highest dispersion (variance over mean) with respect to the genes with the same average expression. This selection was performed using the *scanpy* python package [73]. This analysis was performed separately on genes and microRNAs since they are provided by different experiments and different normalization. We selected in this way 3000 genes and ∼1200 miRNAs.

Furthermore, we applied a standard approach to reduce the weights of the links and applied a log(FPKM+1) transformation to the data before running the topic models. This helped us to reduce by some order of magnitudes the number of edges (as we mentioned above, in this class of algorithms, the weight of a link is mimed adding multiple edges with weight 1) and the model ran several times faster.

In the Copy Number Variation analyses, we chose ∼1300 genes with an average CNV >3.5.

An interesting feature of the SBM type of algorithm is that they are typically robust with respect to gene selection. In the analyses of this paper, we considered only highly variable genes; however, in the supplementary material of [3], we discussed different types of gene selections showing that they were typically leading to similar performances.

In the analysis of the METABRIC dataset, we utilized the previously selected genes and microRNA.

### 4.6. Evaluation Metrics

To evaluate the agreement between the sample partitions and the annotations, we chose the so-called “Normalized Mutual Information” (NMI), which was proposed in [40], in a new evaluation framework for topic models. Moreover, as discussed in [3], it can be shown that NMI is the harmonic average of two metrics that evaluate, respectively, the completeness and the homogeneity of a partition of annotated samples [39]. A cluster is complete if all samples with a given label are assigned to the same cluster; a partition is homogeneous if, in a cluster, all the samples have the same annotation. In order to correctly identify the cancer subtype of a given sample, one would prefer to have a partition in clusters that is both homogeneous and complete.

The NMI is estimated using Shannon’s entropy formula to measure the quantity of information in the partition. The problem of this measure is that even in a random partition, there is a residual entropy and the NMI is not zero; this effect is particularly important in the layers of the models with high resolution (many clusters). In order to avoid this bias, we evaluated this default NMI by randomizing the subtype annotations of the samples. This was performed multiple (∼50) times, each time preserving the number of clusters and the number of samples in every cluster; we call the average NMI on these multiple random assignments $NMI^{*}$; this is the residual information on the considered partition. In the results, we reported *NMI/NMI*∗, which measures how much information the model learns with respect to a random assignment. It is important to stress that this measure has no absolute value and should not be used to compare performances on different datasets; however, it can be successfully used to compare different algorithm in the same dataset, which is what we did in the Results section.

#### Description Length Σ How Well the Model Describes the Data

In addition to the NMI, it is also possible to compare different classes of topic modeling algorithms on their ability to compress the data [65,74]. This can be addressed measuring the description length Σ of a model, which represents, in nat units, the number of bits a model requires to describe the data network. Unlike NMI, it has the advantage not to rely on any ground truth. Using $\frac{\Sigma }{E}$ (where *E* is the total number of edges), it is possible to measure the quantity of information that the model requires to describe an edge. In the models of Figure 5, hSBM requires a $\frac{\Sigma }{E}∼6,26$, which is greater than the 1,4 units required by triSBM. One can estimate the difference of the two $\Delta \left(\frac{\Sigma }{E}\right)≃4,9$; this can be related to the Bayes factor [75] (being the posterior P=exp−Σ) $\Lambda =\exp\Delta \Sigma ≃e^{4,9}≃10^{2,1}$, meaning that the model with miRNA is a ∼100 times more probable description of the data network links. The description lengths of the tetrapartite model and the model with regulatory network are reported in Supplementary Materials (see Figure S6).

### 4.7. Construction of the $\bar{P}\left(\text{Topic}|\text{Subtype}\right)$ Distributions

From the P(topic|sample) distributions, it is easy to obtain the probability P(topic|subtype) by averaging over all samples belonging to the same subtype.

Then, by subtracting to P(topic|subtype) the mean value over the whole dataset, we find a new set of quantities that we define as “centered” distributions (we already used them in [3]; they have the same meaning of the normalized value of the mixture proportion τ in [23])

(5) $$\bar{P}\left(\mathrm{topic}|\mathrm{sample}\right)=P\left(\mathrm{topic}|\mathrm{sample}\right)-\frac{1}{R}\Sigma_{s\in \mathrm{samples}}P\left(\mathrm{topic}|s\right),$$

where *R* is the total number of samples. This procedure can be implemented separately both on the miRNA-topic and on the gene-topic side. The centered P(topic|sample) can be represented as box plots, after grouping samples by their subtype. Examples of these are the box plots reported in Figure 6 on the gene side and Figure 7 on the miRNA side.

### 4.8. Survival Analysis

We performed the survival analyses fitting a COX [58] model.

Our analysis began with the list of the mixtures P(topic|sample). We cleaned up the stages’ labels, removing any additional letter (e.g., *stage ia* became *stage i*), and ended up with four stages: *i*, *ii*, *iii*, and *iv*.

Using Genomic Data Commons tools, we downloaded TCGA metadata and, in particular, *demographic.vital_status*, *demographic.days_to_last_follow_up*, *demographic.days_to_death*, *demographic.gender*, and *diagnoses.age_at_diagnosis*. We estimated the lifetime or the number of days the patient survived after the diagnosis, using *days_to_last_follow_up* if the patient was *Alive* and *days_to_death* for *Dead* patients. A similar approach was recently utilized by [76].

In order to estimate whether a topic is upregulated in a patient, we evaluated the 35th percentile of P(sample|topic) and considered it as a threshold thr. Then, we engineered a feature as follows:

(6) $$up\left(\mathrm{sample}\right)=\left\{\begin{array}{cc} 1 & P(\mathrm{topic}|\mathrm{sample})>thr\\ 0 & P(\mathrm{topic}|\mathrm{sample})\leq thr \end{array}\right.$$

We used these data to fit the hazard with a *COX* model. These analyses were performed using the *lifelines* Python package [77] and, in particular, the COXPHFitter module. We used the lifetime, vital status, and the new feature as input for the fit function.

The Cox model quantified how the topic of miRNAs regulation affected the survival probability. Cox fits the hazard function conditioned to a variable $h\left(t|x\right)=b_{0}\left(t\right)*e^{\Sigma_{i=0}^{n}b_{i}*\left(x_{i}-{\bar{x}}_{i}\right)}$. x is the vector of the *n* covariates considered. The hazard is defined as the ratio of the derivative of the survival and the survival itself $h\left(t\right)=\frac{-S^{\prime }\left(t\right)}{S(t)}$. S(t) is the probability of being alive at time *t*, namely, the number of patients alive at time *t* divided by the total number of patients. The package estimated the ratio between the hazard of samples with topic upregulated and hazard of samples with topic not upregulated. Therefore, we were able to estimate the exp(coef) or hazard ratio $\exp\left(coef\right)=\frac{\mathrm{hazard}\;\mathrm{of}\;\mathrm{samples}\;\mathrm{with}\;\mathrm{topic}\;\mathrm{up}\text{-}\mathrm{regulated}}{\mathrm{hazard}\;\mathrm{of}\;\mathrm{samples}\;\mathrm{with}\;\mathrm{topic}\;\mathrm{not}\;\mathrm{up}\text{-}\mathrm{regulated}}$. Note that the *coef* does not depend on time but is a sort of weighted average of period-specific hazard ratios.

### 4.9. Code and nSBM Software Package

The Python package to run nSBM [25] can be downloaded from GitHub ( https://github.com/BioPhys-Turin/nsbm, accessed on 10 February 2022) or, alternatively, can be installed using Anaconda (https://anaconda.org/conda-forge/nsbm, accessed on on 10 February 2022) by running conda install nsbm -c conda-forge.

We discussed in this paper the application using genomics data; however, the package is written in a way that makes it agnostic with respect to the type of data it receives in input and to the number of branches. One can ideally integrate as many different sources (’omics) of data as needed. Eventually, it can process not only biological data, but every kind of dataset whose input could be represented as a rectangular matrix (Bag of Words) for each feature.

## 5. Conclusions

In conclusion, the nSBM model we propose here, integrating multiple sources of information into an hSBM analysis, should be useful to extract a lot of information from transcriptomics data.

- Using the python package: *nSBM*, inherited from hSBM [10], ready to install and easily executable on n-partite networks, will be straightforward to address different types of biological data.
- Second, the integration of multiple sources of data, such as microRNA expression levels and the protein-coding mRNA ones, greatly improves the ability of the algorithm to identify breast cancer subtypes.
- Third, we use our results to identify a few genes and miRNAs and characterize a few chromosomal duplications that seem to have a particular prognostic role in breast cancer and could be used as signatures to predict the particular breast cancer subtypes.

In conclusion, this paper released a new tool to easily integrate different sources of data into a topic-modeling analysis.

We showed some application in a specific case (breast cancer) with some sources of data (mRNA, miRNA, CNV). Indeed, this approach can be applied to other datasets and, more importantly, to any possible sources of data (genomics, proteomics, lncRNA, circRNA…).

## Figures and Tables

**Table 1:** GSEA FDR enrichment *P*-values on the gene-topics. For each gene-topic, only the terms with the strongest enrichment are reported. In brackets is the number of genes in each set (topic). Lists are available at https://github.com/BioPhys-Turin/keywordTCGA/blob/main/brca/trisbm/trisbm_level_0_topics.csv, accessed on 10 February 2022.

Term	False Discovery Rate
gene-topic 6 (55)	
SMID_BREAST_CANCER_BASAL_DN	8.14×$\;10^{-22}$
FARMER_BREAST_CANCER_APOCRINE_VS_LUMIN MINAL	3.67×$\;10^{-7}$
gene-topic 8 (19)	
MODULE_54 (cell cycle)	2.31×$\;10^{-20}$
gene-topic 12 (13)	
MODULE_1 (ovary genes)	1.52×$\;10^{-7}$
SMID_BREAST_CANCER_BASAL_DN	$1.53\times 10^{-7}$
gene-topic 15 (26)	
HALLMARK_EPITHELIAL_MESENCHYMAL_TRANSINSITION	7.93×$\;10^{-15}$
gene-topic 25 (40)	
CHARAFE_BREAST_CANCER_LUMINAL_VS_MESEN SENCHYMAL_UP	4.59×$\;10^{-15}$
SMID_BREAST_CANCER_BASAL_DN	3.98×$\;10^{-14}$
VANTVEER_BREAST_CANCER_ESR1_UP	7.91×$\;10^{-5}$
gene-topic 27 (44)	
SMID_BREAST_CANCER_ERBB2_UP	1.73×$\;10^{-7}$
gene-topic 28 (53)	
SMID_BREAST_CANCER_BASAL_UP	8.11×$\;10^{-23}$
gene-topic 37 (54)	
FAN_OVARY_CL13_MONOCYTE_MACROPHAGE	1.36×$\;10^{-14}$
VANTVEER_BREAST_CANCER_ESR1_DN	7.63×$\;10^{-11}$
gene-topic 44 (37)	
SMID_BREAST_CANCER_BASAL_UP	3.22×$\;10^{-13}$
gene-topic 53 (39)	
SMID_BREAST_CANCER_BASAL_DN	4.46×$\;10^{-11}$
gene-topic 55 (58)	
SMID_BREAST_CANCER_BASAL_DN	4.96×$\;10^{-33}$
FARMER_BREAST_CANCER_BASAL_VS_LULMINAL	1.21×$\;10^{-14}$
VANTVEER_BREAST_CANCER_ESR1_UP	2.91×$\;10^{-14}$
SMID_BREAST_CANCER_LUMINAL_B_UP	7.33×$\;10^{-12}$
gene-topic 68 (54)	
SMID_BREAST_CANCER_BASAL_UP	6.19×$\;10^{-21}$
CHARAFE_BREAST_CANCER_LUMINAL_VS_BASAL_DN	1.32×$\;10^{-14}$
SMID_BREAST_CANCER_LUMINAL_B_DN	1.46×$\;10^{-14}$
CHARAFE_BREAST_CANCER_LUMINAL_VS_MESENCHYMAL_DN	3.15×$\;10^{-10}$

**Table 2:** **GSEA results on the miRNA-topics.** We selected and reported the ones with the strongest enrichment. Lists are available at https://github.com/BioPhys-Turin/keywordTCGA/blob/main/brca/trisbm/trisbm_level_1_metadata.csv, accessed on 10 February 2022.

Term	False Discovery Rate
miRNA-topic 7 (57)	
chr14q32	1.81×$\;10^{-29}$
WP_ALZHEIMERS_DISEASE	2.88×$\;10^{-4}$
miRNA-topic 11 (60)	
chr19q13	1.57×$\;10^{-6}$

**Table 3:** microRNAs sorted by their P(miRNA|miRNA-topic7). The most important miRNAs in our candidate miRNA-topic. Most of them are well-known in literature. The complete list is available at https://github.com/BioPhys-Turin/keywordTCGA/blob/main/brca/trisbm/trisbm_level_1_keyword-dist.csv, accessed on 10 February 2022.

microRNA	*P*(miRNA|miRNA-topic 7)
hsa-mir-654	0.047
hsa-mir-758	0.046
hsa-mir-493	0.042
hsa-mir-889	0.041
hsa-mir-34c	0.041
hsa-mir-431	0.039
hsa-mir-369	0.039
hsa-mir-370	0.039
hsa-mir-410	0.037
hsa-mir-154	0.035
hsa-mir-495	0.035
hsa-mir-511	0.035
hsa-mir-411	0.033
hsa-mir-432	0.032
hsa-mir-31	0.031
hsa-mir-487b	0.030
hsa-mir-376c	0.030
hsa-mir-412	0.030
…	<0.030

**Table 4:** Enrichment analysis on the Copy Number Variation branch of tetraSBM. All the lists are available at https://github.com/BioPhys-Turin/keywordTCGA/blob/main/brca/tetrasbm/trisbm/trisbm_level_0_kind_3_metadata.csv, accessed on 10 February 2022.

Term	False Discovery Rate
CNV-topic 1 (41)	
chr20q13	3.66×$\;10^{-63}$
NIKOLSKY_BREAST_CANCER_20Q12_Q13_AMPLICON	3.66×$\;10^{-63}$
CNV-topic 3 (50)	
chr17q23	1.42×$\;10^{-60}$
NIKOLSKY_BREAST_CANCER_17Q21_Q25_AMPLICON	1.42×$\;10^{-60}$
CNV-topic 4 (17)	
chr8q24	1.×$\;10^{-25}$
NIKOLSKY_BREAST_CANCER_8Q23_Q24_AMPLICON	1.5×$\;10^{-7}$
CNV-topic 6 (53)	
chr8q12	1.02×$\;10^{-37}$
chr8q11	1.4×$\;10^{-24}$
chr8q13	1.×$\;10^{-19}$
NIKOLSKY_BREAST_CANCER_8Q12_Q22_AMPLICON	1.16×$\;10^{-8}$
CNV-topic 7 (47)	
chr1q32	2.07×$\;10^{-50}$
chr1q41	2.06×$\;10^{-27}$
CNV-topic 13 (14)	
chr8q11	4.6×$\;10^{-28}$
NIKOLSKY_BREAST_CANCER_8P12_P11_AMPLICON	3.12×$\;10^{-27}$
CNV-topic 18 (16)	
NIKOLSKY_BREAST_CANCER_17Q21_Q25_AMPLICON	1.1×$\;10^{-29}$
chr17q23	3.2×$\;10^{-21}$
CNV-topic 22 (11)	
chr8p11	2.21×$\;10^{-25}$
NIKOLSKY_BREAST_CANCER_8P12_P11_AMPLICON	1.03×$\;10^{-20}$
CNV-topic 25 (11)	
NIKOLSKY_BREAST_CANCER_8P12_P11_AMPLICON	5.21×$\;10^{-28}$
chr8p11	1.1×$\;10^{-25}$
CNV-topic 26 (21)	
chr20q13	1.62×$\;10^{-38}$
NIKOLSKY_BREAST_CANCER_20Q12_Q13_AMPLICON	1.66×$\;10^{-34}$
CNV-topic 28 (5)	
NIKOLSKY_BREAST_CANCER_17Q11_Q21_AMPLICON	1.17×$\;10^{-8}$
chr17q21	2.96×$\;10^{-6}$

## Data Availability

Notebooks to reproduce the results in this work are available on GitHub at https://github.com/BioPhys-Turin/keywordTCGA, accessed on 10 February 2022).

## Supplementary Materials

The following supporting information can be downloaded at: https://www.mdpi.com/article/10.3390/cancers14051150/s1, Figure S1: Normalized Mutual information of hSBM and triSBM partition compared with the Breast Cancer Consensus Subtypes of Ref. [41]. Figure S2: Validation on METABRIC dataset. Figure S3: Multivariate (Log) Hazard Ratios. Figure S4: Normalized Mutual Information of models with samples and mRNA (hSBM), miRNA (triSBM) and mRNA, and both miRNA and CNV (tetraSBM). Adding CNV introduces noise to the model. Figure S5: Normalized Mutual Information of bipartite models with samples and mRNA (hSBM) and samples with Copy Number Variation (CNV). Adding CNV introduces noise to the model. Figure S6: Description length of different settings. Figure S7: Days of survival of different patients in clusters.

## Abbreviations

The following abbreviations are used in this manuscript:

SBM	Stochastic Block Modeling
TCGA	The Cancer Genome Atlas
GSEA	Gene Set Enrichment Analysis
FDR	False Discovery Rate
FPKM	Fragments Per Kilobase of transcript per Million mapped reads
